# A 3-Year-Old Child With Incidental High-Dose Vitamin D Intoxication: A Case Report and Literature Review

**DOI:** 10.1155/crie/6614996

**Published:** 2025-10-21

**Authors:** Hossein Kasiri, Amir Hasan Farzaneh, Navid Khosravi

**Affiliations:** ^1^Department of Clinical Pharmacy, Faculty of Pharmacy, Mazandaran University of Medical Sciences, Sari, Iran; ^2^Department of Toxicology and Forensic Medicine, Imam Khomeini Hospital, Mazandaran University of Medical Sciences, Sari, Iran

**Keywords:** cholecalciferol, ergocalciferol, hypervitaminosis D, pediatrics, toxicity, vitamin D

## Abstract

Although rare, vitamin D toxicity is a serious condition that commonly results from excessive self-medication. Clinical manifestations range from asymptomatic hypercalcemia to severe, life-threatening complications. We present the case of a previously healthy 3-year-old child who accidentally ingested 800,000 IU of vitamin D3 (cholecalciferol) and remained asymptomatic. Management included intravenous hydration, oral prednisolone, and strict dietary calcium restriction, alongside regular monitoring of serum calcium and vitamin D levels. The patient was discharged after 11 days and showed normalized laboratory parameters during a 3-month follow-up. This case underscores the importance of early diagnosis, immediate cessation of vitamin D intake, and conservative medical management in preventing complications. It also highlights the critical need for public education regarding the safe use of dietary supplements, particularly in pediatric populations.

## 1. Introduction

Vitamin D toxicity most frequently results from the unintentional or inappropriate ingestion of supplements. Without prompt diagnosis, it can lead to severe complications, primarily due to resultant hypercalcemia. Hypervitaminosis D may be caused by excessive intake of vitamin D itself, its metabolites (25-hydroxyvitamin D or 1,25-dihydroxyvitamin D), or the topical application of potent analogs. Toxicity generally manifests when daily doses exceed 10,000 IU and serum 25-hydroxyvitamin D (25[OH]D) levels surpass 150 ng/mL [1, 2].

Symptoms are typically nonspecific and secondary to hypercalcemia, ranging from neuropsychiatric disturbances (e.g., confusion, psychosis, stupor, or coma) to gastrointestinal (e.g., abdominal pain, vomiting, polydipsia, anorexia, constipation, and pancreatitis), cardiovascular, and renal complications (e.g., hypercalciuria, acute kidney injury, dehydration, and nephrocalcinosis) [3–5].

We report a pediatric case of significant vitamin D intoxication following the accidental ingestion of 800,000 IU of cholecalciferol, which was managed successfully with conservative treatment.

## 2. Case Presentation

A 3-year-old boy was brought to the poisoning emergency department of Razi Hospital, Qaemshahr, Iran, on October 13, 2022, after accidentally consuming approximately 16 pearls of vitamin D3 (50,000 IU each), totaling 800,000 IU. He had no significant prior medical history, and his mother denied any regular vitamin D supplementation before this incident.

On examination, the patient was clinically stable and asymptomatic. Vital signs were as follows: heart rate 105 bpm, blood pressure 95/65 mm Hg, respiratory rate 23 breaths/min, temperature 36.2°C, and oxygen saturation 98% on room air. Initial management included intravenous hydration with isotonic saline and blood sampling for routine laboratory tests and plasma 25 (OH)D level measurement. An electrocardiogram revealed a normal sinus rhythm.

The initial laboratory data on admission are presented in Table 1. Treatment was initiated with intravenous isotonic saline hydration and oral prednisolone (7.5 mg twice daily). A diet restricted to less than 400 mg of calcium per day was implemented. On the third hospital day, furosemide (0.3 mg/kg/day) was added to the regimen based on trending vitamin D and calcium levels.

Serial biochemical monitoring during hospitalization is detailed in Table 2. Although the patient remained clinically asymptomatic, laboratory findings revealed intermittent hypercalcemia, with corrected calcium peaking at 11.26 mg/dL on Days 4 and 6. The serum 25 (OH)D level peaked at 880 ng/mL on Day 5. All parameters improved with conservative management.

The patient was discharged after 11 days with strict instructions to maintain adequate hydration, adhere to a low-calcium diet (<400 mg/day), avoid direct sun exposure, and refrain from taking any supplements containing vitamin D or calcium. During follow-up, a steady decrease in vitamin D levels was observed, and the patient did not develop symptoms of hypercalcemia (Table 3).

## 3. Discussion and Review of Literature

For this literature review, relevant English-language articles were identified via PubMed using the keywords “vitamin D toxicity,” “hypervitaminosis D,” “child,” and “infant.“

Vitamin D is essential for maintaining calcium–phosphate homeostasis and is primarily used to prevent conditions like rickets and osteoporosis [6]. The recommended dietary allowance (RDA) for vitamin D is 400 IU for infants aged 0–12 months, 600 IU for individuals aged 1–70 years (including during pregnancy and lactation), and 800 IU for adults over 70 [7, 8]. The precise threshold for toxicity is not well-defined; however, it typically requires sustained daily intake exceeding 100,000 IU for at least 1 month [6, 9].

Our patient ingested a single dose approximately 1300 times the RDA for his age. Vitamin D intoxication can be caused by the parent compound or its metabolites. Due to the lipophilic nature of cholecalciferol and its storage in adipose tissue, the risk of toxicity can theoretically persist for months. However, the duration of clinically significant hypercalcemia is often shorter with appropriate intervention. In our case, 25(OH)D levels normalized within 3 months, consistent with its half-life of approximately 15 days. The prolonged half-life of the parent compound likely explains the extended period of detectable 25 (OH)D, but it did not lead to sustained hypercalcemia in this patient [5, 10].

The correlation between the severity of vitamin D intoxication and the degree of hypercalcemia in children remains poorly characterized. In a study by Farnaghi et al. [11] 15 children under 12 years of age (mean ingested dose 406,700 ± 227,400 IU) presented to a pediatric emergency room; only one child developed hypercalcemia. This suggests that acute vitamin D poisoning may be a relatively benign condition among Iranian children, possibly due to the high prevalence of underlying vitamin D deficiency in this population. This may explain why our patient, despite a massive overdose, did not develop severe hypercalcemia.

Hypercalcemia is categorized as mild (corrected calcium < 12 mg/dL), moderate (12–14 mg/dL), or severe (>14 mg/dL) [12]. It typically manifests with symptoms, such as anorexia, nausea, vomiting, constipation, abdominal pain, muscle weakness, polyuria, and dehydration. In our case, despite the high dose, serum calcium remained in the mild range, and the patient was asymptomatic. There are no standardized treatment guidelines for vitamin D toxicity in children; management relies on clinical judgment and evidence from case reports and small-scale studies [13].

The cornerstone of managing vitamin D toxicity is the immediate identification and discontinuation of all vitamin D sources. First-line treatment strategies include hydration with isotonic saline, glucocorticoids, a low-calcium diet, and, in symptomatic cases, loop diuretics to enhance calciuresis. Bisphosphonates, which induce osteoclast apoptosis and inhibit bone resorption, are effective for managing hypercalcemia. The use of alendronate for treating vitamin D intoxication in infants was first reported in 2003 [14]. In a study by Sezer et al. [15] alendronate normalized calcium levels four times faster than prednisolone and reduced the length of hospital stay. Second-line treatments include phenobarbital, ketoconazole, aminoquinolines (e.g., chloroquine and hydroxychloroquine), and specific CYP27B1 (1 α-hydroxylase) inhibitors [5, 16, 17].

In our case, the patient's early presentation, prompt initiation of hydration and glucocorticoids, and severe restriction of dietary vitamin D and calcium likely prevented complications, making more aggressive therapies unnecessary. Throughout the 11-day hospitalization, the patient maintained high vitamin D levels but remained asymptomatic, obviating the need for additional treatments. The patient was discharged after 11 days with strict instructions to maintain adequate hydration, adhere to a low-calcium diet (<400 mg/day), avoid sun exposure, and avoid any supplements containing vitamin D or calcium.

The patient was followed for 3 months. By the second month, his 25(OH)D level had decreased below 100 ng/mL and normalized by the end of the follow-up period.

## 4. Conclusion

Vitamin D toxicity is often a consequence of unintentional or excessive oral supplementation. This case demonstrates that even with extremely high-dose exposure, early identification, prompt intervention, and close clinical follow-up can prevent complications. Public education and increased awareness regarding the potential dangers of supplement misuse are essential, particularly in households with young children. Individuals administering vitamin D supplements, especially at doses exceeding age-appropriate recommendations, should exercise caution and seek medical supervision.

## Figures and Tables

**Table 1:** First day laboratory data.

Variable	Reference range, in hospital	Day of first admission	1 Week after first admission
Hemoglobin (g/dL)	13–17	13.5	
White blood cells (per µL)	4000–11,000	6700	
Platelet count (per µL)	150,000–450,000	370,000	
Sodium (meq/L)	135–145	141	143
Potassium (meq/L)	3.5–5.5	4.5	5.6
25 (OH)D (ng/mL)	Deficient: <20, insufficient: 21–29, normal: 30–100, toxic: >100	116.6	710
Albumin (g/dL)	3.8–5.1	4.7	4.8
Magnesium (mg/dL)	1.9–2.5	1.9	2
PTH (pg/mL)	12–65	12.3	
Creatinine (mg/dL)	0.7–1.4	0.6	0.5
Glucose (mg/dL)	65–99	93	
Random urine Ca (mg/dL)	0.9-37.9		7.8
Ca/Cr ratio	<0.15		0.13
Random urine creatinine (mg/dL)	24–392		60

**Table 2:** Serial biochemical parameters during hospitalization.

Date	Hospital day	Corrected calcium (mg/dL)	25 (OH)D (ng/mL)	Phosphorus (mg/dL)
October 13, 2022	Day 1	8.84	116.6	5.4
October 14, 2022	Day 2	9.82		5.8
October 15, 2022	Day 3	9.36	780	6
October 16, 2022	Day 4	11.26		5.5
October 17, 2022	Day 5	10.76	880	5.3
October 18, 2022	Day 6	11.26		5.5
October 19, 2022	Day 7	9.96	805	5.8
October 20, 2022	Day 8	10.6		4.5
October 21, 2022	Day 9	9.36	710	5.5
October 22, 2022	Day 10	9.56		5.3
October 23, 2022	Day 11	9.76	680	5
October 24, 2022	Discharge			

*Note:* reference ranges: corrected calcium, 8.1–10.3 mg/dL; phosphorus, 3.2–7.7 mg/dL; 25 (OH)D, normal 30–100 ng/mL, toxic > 100 ng/mL.

**Table 3:** Vitamin D and calcium levels of patients after discharge (follow-up).

Month	25(OH)D (ng/mL)	Calcium (corrected, mg/dL)^a^
November	267.7	8.7
December	65.8	9.04
January	42.5	9.12

^a^Corrected calcium (mg/dL) = (0.8 × (normal albumin − Pt's albumin)) + serum Ca.

## Data Availability

The data that support the findings of this study are available on request from the corresponding author. The data are not publicly available due to privacy or ethical restrictions.
